# Fetal pneumonia and polyarthritis associated with *Ureaplasma diversum* infection in an aborted beef cattle fetus in Brazil

**DOI:** 10.1007/s11259-026-11476-x

**Published:** 2026-08-27

**Authors:** Letícia Perri Luciano, Roger Fernando Neves da Silva, Wuglenya Daislla Martins da Silva, Ícaro Guilherme dos Santos, Bruna Souza Serrano, Gabriela Viana Castilho Bichara, Maria Sinara Matos, Fabricio Andrade Gomes, Nataliê Ecker, Tayane Bruna Soares Magalhães, Marcos de Almeida Souza, Daniel Moura de Aguiar, Caroline Argenta Pescador

**Affiliations:** 1 Laboratory of Veterinary Pathology, Federal University of Mato Grosso (UFMT), Cuiabá, Mato Grosso Brazil; 2 Laboratory of Vector-Borne Rickettsiosis, Faculty of Veterinary Medicine, Federal University of Mato Grosso (UFMT), Cuiabá, Brazil; 3 Faculty of Veterinary Medicine, Laboratory of Veterinary Pathology, Federal University of Mato Grosso, Av. Fernando Corrêa da Costa, 2367 - Boa Esperança, Cuiabá, MT 78060-900 Brazil

**Keywords:** Bovine abortion, Fetal arthritis, Mollicutes

## Abstract

*Ureaplasma diversum* is an opportunistic bovine pathogen associated with reproductive disorders, including infertility, granular vulvovaginitis, placentitis, and abortion; however, detailed descriptions of fetal osteoarticular lesions remain exceptionally uncommon. This report describes the gross, histopathological, and molecular findings of a bovine abortion associated with *U. diversum* infection presenting with concurrent pulmonary and articular lesions, representing an uncommon manifestation in bovine fetuses in Brazil. An approximately six-month-old Angus fetus was submitted for complete postmortem examination following abortion. Tissue samples were collected for histopathology and molecular investigation of major infectious agents associated with bovine reproductive failure. Gross examination revealed bilateral coxofemoral joint lesions characterized by articular cartilage irregularity and erosions. Histologically, the lungs exhibited moderate multifocal pyogranulomatous pneumonia with prominent bronchus-associated lymphoid tissue (BALT) hyperplasia. The coxofemoral joints showed severe multifocal subacute erosive fibrinonecrotizing arthritis with proliferative lymphoplasmacytic synovitis and secondary osteonecrosis. Histologically, fibrovascular pannus formation, cartilage necrosis, and extension of the lesion into the subchondral bone were observed. Quantitative PCR detected *U. diversum* DNA in brain, spleen, thymus, liver, and kidney, whereas the paraffin-embedded coxofemoral joint sample tested negative. Molecular assays for other major abortifacient pathogens, including bovine viral diarrhea virus (types 1, 2, and 3), bovine herpesvirus 1 and 6, *Leptospira* spp., *Brucella abortus*, *Mycoplasma bovis*, and *Neospora caninum* were negative. The exclusion of other common infectious causes, combined with the molecular confirmation of *U. diversum* and the characteristic inflammatory lesions, support an etiological association between *U. diversum* infection and the abortion. These findings expand the pathological spectrum of *U. diversum* infection in bovine fetuses, particularly regarding osteoarticular involvement, and emphasize the diagnostic value of systematic joint evaluation combined with molecular testing in bovine abortion investigations.

## Introduction

*Ureaplasma diversum* is a bacterium belonging to the family Mycoplasmataceae, order Mycoplasmatales, and class Mollicutes. It is an opportunistic pathogen frequently identified in the respiratory and reproductive tracts of cattle. The microorganism was first isolated in 1967 from tissue samples of the vagina, urethra, and bladder collected from clinically healthy cows at slaughterhouses (Santos-Junior et al. 2021; Taylor-Robinson et al. 1967).

In females, *U. diversum* has been associated with severe granular vulvovaginitis, salpingitis, endometritis, mastitis, and placentitis, which may result in abortion or the birth of weak calves (Cardoso et al. 2000; Queiroz-Machado et al. 2026). In bulls, the pathogen has been linked to seminal vesiculitis, balanoposthitis, epididymitis, and other alterations caused by morphological and functional damage to spermatozoa, thereby compromising fertility and overall herd reproductive efficiency (Miller et al. 1994; Eaglesome et al. 1992). Moreover, chronic Granular Vulvovaginitis Syndrome (GVS) caused by *U. diversum*, may ascend to the uterus, progress to endometritis, and ultimately lead to infertility or abortion (Barkallah et al. 2014; Miller et al. 1994).

Brazil has the second-largest cattle herd in the world, emphasizing the economic importance of infectious agents that impair reproductive performance. *Ureaplasma diversum* is widely distributed in cattle populations worldwide and has been associated with reproductive disorders, including pustular vulvovaginitis, repeat breeding, infertility, abortion, and reduced reproductive efficiency (Eaglesome et al. 1992; Mulira et al. 1992; Oliveira Filho et al. 2005; Santos et al. 2013; Buzinhani et al. 2011). In cattle with reproductive disorders, reported detection rates range from 38.8% to 83.8%, with studies from Canada and several Brazilian regions demonstrating the broad distribution of this pathogen (Mulira et al. 1992; Oliveira Filho et al. 2005; Santos et al. 2013; Gaeti et al. 2014; Voltarelli et al. 2018).

Despite its recognized role in reproductive disease, reports describing fetal infection and associated pathological findings remain limited, particularly in beef cattle under Brazilian field conditions. Therefore, this study describes a case of fetal loss associated with *U. diversum* infection accompanied by articular lesions in a bovine fetus. The objectives were to (i) characterize the macroscopic and microscopic lesions observed in the aborted fetus and (ii) highlight the diagnostic tools used for the detection of *U. diversum* in cases of bovine abortion.

## Materials and methods

The aborted bovine fetus was submitted by a field veterinarian from the municipality of Jaciara, Mato Grosso State, Brazil, together with epidemiological data and clinical history. Gestational age was estimated based on crown–rump length (Barr et al. 1990) and complemented by the evaluation of integumentary development, including the presence or absence of hair on the lips, eyebrows, and muzzle, eruption of the incisor teeth, and the presence or absence of horn pits.

Tissue fragments from the brain, lung, heart, spleen, liver, abomasum, lymph nodes, kidney, thymus, thyroid gland, tongue, skeletal muscle, and coxofemoral joint were collected during necropsy and fixed in 10% neutral buffered formalin for routine histopathological processing. For the coxofemoral joint specifically, following fixation, samples were decalcified in a 10% ethylenediaminetetraacetic acid (EDTA, EasyPath, Erviegas) solution (0.1 M phosphate buffer, pH 7.8) for approximately 8–10 weeks, with the solution changed weekly, prior to routine histological processing (Schmitz et al. 2010).

For molecular analysis, samples of the brain, spleen, thymus, liver, and kidney were aseptically collected, with each organ processed individually and placed into a separate sterile microtube. In addition, paraffin-embedded sections of the coxofemoral joint were collected. All samples were submitted to the Laboratory of Virology and Rickettsioses, Federal University of Mato Grosso (UFMT). Polymerase chain reaction (PCR) assays were performed to investigate the presence of major infectious agents associated with bovine reproductive failure, including Bovine viral diarrhea virus (BVDV) types 1, 2, and 3, bovine herpesvirus type 1 (BoHV-1), bovine herpesvirus type 6 (BoHV-6), *Leptospira* spp., *Brucella abortus*, *Mycoplasma bovis*, *Neospora caninum* and *U. diversum*.

The organs were homogenized with beads using a BeadBug™ homogenizer (Benchmark Scientific, Sayreville, NJ, USA) and subjected to genomic DNA/RNA extraction using the MagMAX™ Core Nucleic Acid Purification Kit (Applied Biosystems, Austin, TX, USA), following the manufacturer’s instructions. The paraffin section of coxofemoral joint was subjected to genomic DNA/RNA extraction using the MagMAX™ FFPE DNA/RNA Ultra Kit (Applied Biosystems, Austin, TX, USA), following the manufacturer’s instructions. Genomic material was quantified using a NanoDrop™ Lite Plus spectrophotometer (Thermo Fisher Scientific, Wilmington, DE, USA). Extracted DNA was subsequently analyzed using TaqMan qPCR assays targeting the *glycoprotein B* (*gB*) gene of BoHV-1 (WOAH, 2024), and BoHV-6 (Kubiś et al. 2013), the *Nc5* gene of *Neospora caninum* (Barry et al. 2019), the *IS711* gene of *Brucella abortus* (Bounaadja et al. 2009), the LipL32 gene of *Leptospira* spp. (Miotto et al. 2018), the *oppD* gene of *Mycoplasma bovis* (Goto et al. 2020), and the *16 S RNA* gene of *U. diversum* (Marques et al. 2013), using the GoTaq^®^ Probe qPCR Master Mix (Promega^®^, Madison, WI, USA). BVDV was analyzed separately using a One-Step TaqMan qPCR assay targeting the 5′ untranslated region [5′ UTR] (Mahlum et al. 2002), with the One-Step GoTaq^®^ Probe qPCR Master Mix according to the manufacturer’s instructions (Promega^®^, Madison, WI, USA). Amplification conditions consisted of 45 °C for 10 min (for cDNA synthesis in BVDV assays), followed by an initial denaturation step at 95 °C for 10 min, and 40 cycles of 95 °C for 15 s and 55 °C (for BVDV) or 60 °C (for the remaining agents) for 1 min. All reactions were performed using a QuantStudio™ 3 Real-Time PCR System (Applied Biosystems, Austin, TX, USA). All samples and controls were analyzed in duplicate, and only samples showing consistent amplification in both reactions were considered positive.

Ultrapure water was used as the negative control for both DNA extraction and qPCR assays. Positive controls for the remaining pathogens consisted of commercially available vaccines or DNA extracted from clinical cases previously confirmed at the Veterinary Pathology Laboratory. For *U. diversum*, the positive control was DNA obtained from the clinical case described by Ribeiro et al. (2025).

## Results

According to the clinical history, a single abortion was recorded in an approximately 8-year-old Angus beef cow. The farm maintained a herd of approximately 2,700 cattle, and the sanitary protocol included routine vaccination against infectious bovine rhinotracheitis (IBR), bovine viral diarrhea virus (BVDV), and leptospirosis.

The fetus was a female Angus calf in good preservation condition, weighing 8 kg and measuring 56 cm from the caudal aspect of the eye to the tail insertion, corresponding to approximately six months of gestational age. The placenta was not submitted for examination.

**Fig. 1:**
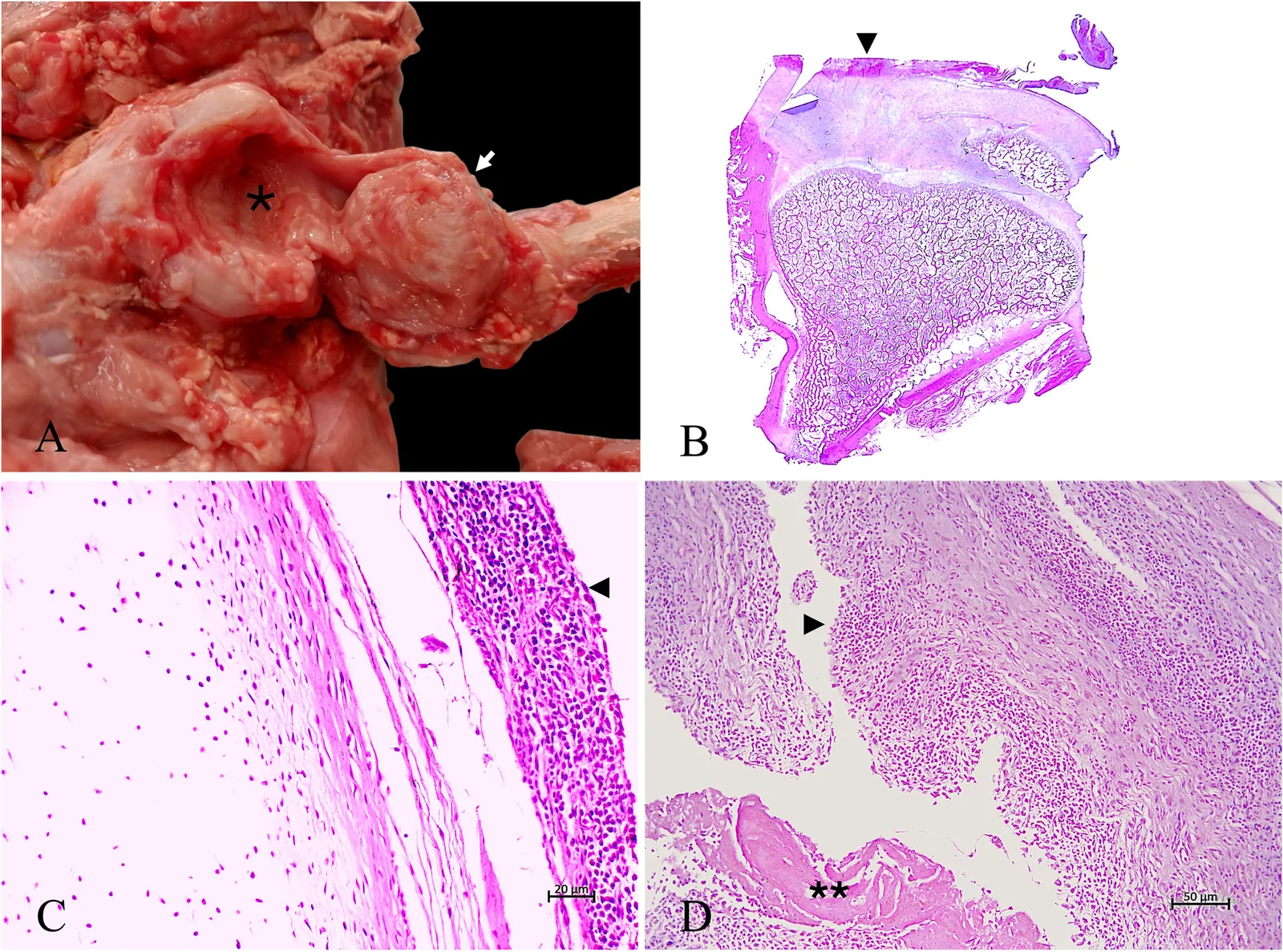
Joint lesions in an aborted bovine fetus (6 months gestation) associated with *Ureaplasma diversum*. (**A**) Coxofemoral joint. The acetabular surface is reddened, thickened, and irregular (black asterisk), and the femoral articular cartilage is reddened, roughened, and friable (white arrow). (**B**) Subgross image of the proximal femoral region showing fibrovascular pannus extending over and infiltrating the articular cartilage (black arrowhead), accompanied by mononuclear inflammatory infiltrates. (**C**) Synovial membrane. Dense thickening is observed, associated with an inflammatory infiltrate composed of lymphocytes and plasma cells (black arrowhead). H&E, 20 μm. (**D**) Synovial membrane. Proliferative lymphoplasmacytic synovitis with reactive villous projections (black arrowhead). Double asterisks indicate subchondral osteonecrosis. H&E, 50 μm

At necropsy, the lungs were atelectasic. Both coxofemoral joints exhibited irregular thickening of the articular cartilage with multifocal bilateral erosions. The acetabular fossa was opaque, irregular, and deepened, indicating loss of normal articular cartilage (Fig. 1a). Microscopically, the proximal femoral articular cartilage was overlain by a focally extensive mononuclear inflammatory infiltrate extending along the cartilaginous surface and partially replacing the superficial cartilage layer, resulting in erosive cartilage damage (Fig. 1b). The synovial membrane was markedly and diffusely thickened by dense lymphoplasmacytic inflammatory infiltrates and mild edema, consistent with proliferative lymphoplasmacytic synovitis (Fig. 1c). Multifocally, fibrovascular tissue infiltrated areas of necrotic cartilage and extended over the articular surface, forming fibrovascular pannus with reactive villous synovial projections associated with marked lymphoplasmacytic inflammation and fewer intact and degenerate neutrophils (Fig. 1d). In severely affected areas, the lesion extended into the subchondral bone, resulting in osteonecrosis. Overall, these findings were consistent with severe multifocal subacute erosive fibrinonecrotizing arthritis with proliferative lymphoplasmacytic synovitis and secondary osteonecrosis. Additionally, the lungs displayed multifocal alveolar and septal inflammatory infiltrates composed mainly of lymphocytes, plasma cells, and macrophages, with fewer neutrophils. BALT hyperplasia, multinucleated cells and keratin filaments were also observed, findings consistent with moderate multifocal pyogranulomatous pneumonia.

**Fig. 2:**
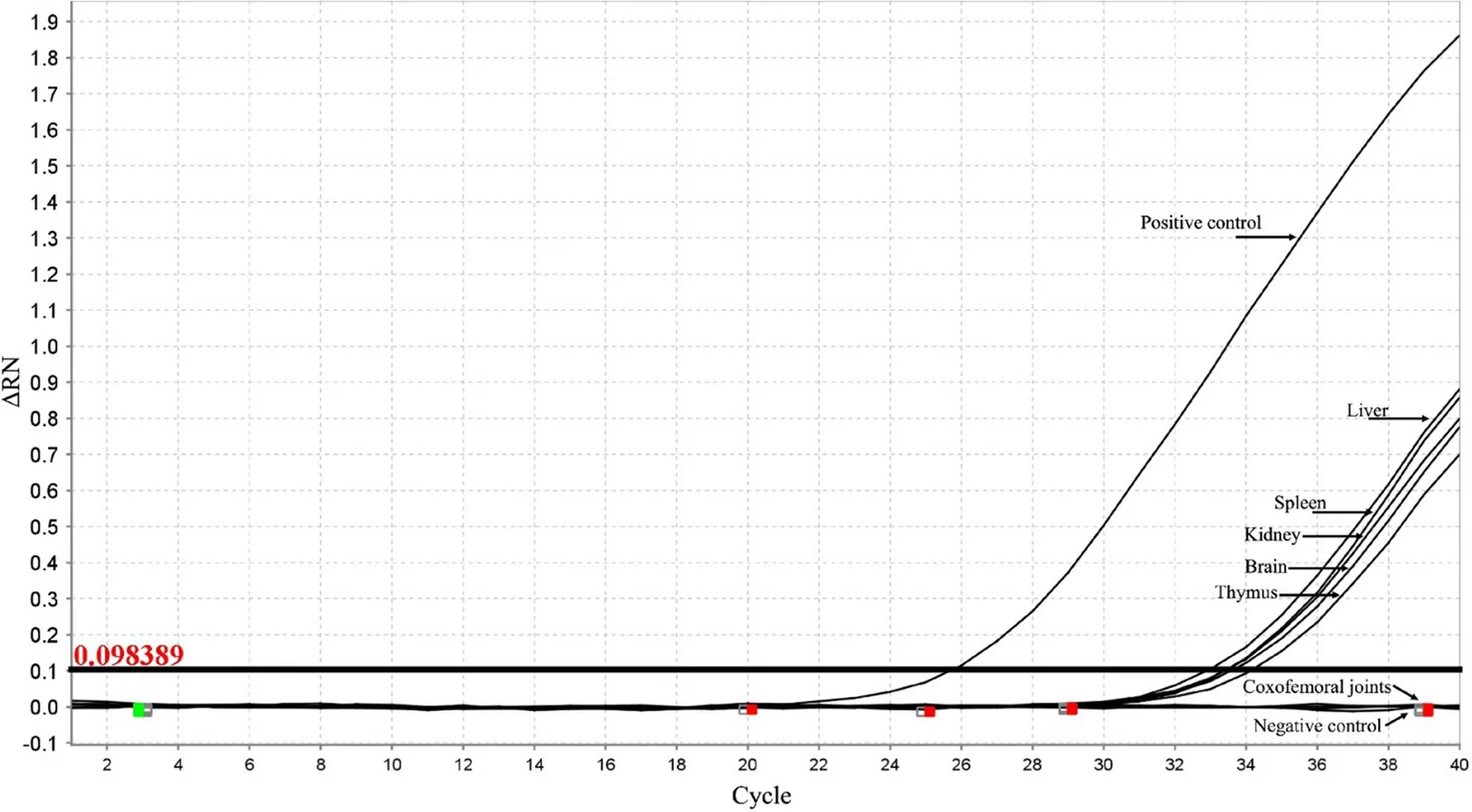
Representative amplification plot of the *Ureaplasma diversum*-specific TaqMan qPCR assay. The positive control amplified at Ct 25.6, whereas DNA extracted from fetal tissues yielded Ct values ranging from 32.9 to 34.1. No amplification was observed for the negative extraction control or the paraffin-embedded coxofemoral joint sample

The fetus tested positive for *U. diversum* by qPCR in the brain, spleen, thymus, liver, and kidney, whereas the paraffin-embedded coxofemoral joint sample tested negative. The cycle threshold (Ct) values were 32.9, 33.3, 33.3, 33.5 and 34.1 respectively (Fig. 2). Additional PCR assays for BVDV-1, 2 and 3, BoHV-1, BoHV-6, *Leptospira* spp., *B. abortus*, *M. bovis* and *N. caninum* were negative.

## Discussion

This case describes the gross, histopathological, and molecular findings of a bovine abortion associated with osteoarticular lesions and pneumonia in which *U. diversum* was detected by PCR. The consistent detection in multiple fetal organs further supports that the positive qPCR results were not attributable to sporadic contamination during sample processing. Although this microorganism has been increasingly recognized as a relevant reproductive pathogen implicated in bovine abortion worldwide (Santos-Junior et al. 2021), reports describing associated osteoarticular lesions remain limited, highlighting the relevance of the present findings for expanding the currently recognized pathological spectrum of infection.

Abortion associated with *U. diversum* has been predominantly reported during the final third of gestation, whereas earlier infections may result in embryonic loss that frequently goes unnoticed (Ruhnke et al. 1984). The estimated gestational age of approximately six months in the present fetus is therefore consistent with previously reported patterns of disease manifestation.

The primary route of transmission of *U. diversum* is sexual, with infected bulls acting as important reservoirs within herds. Additionally, assisted reproductive technologies, including artificial insemination and embryo transfer, have been described as potential routes of dissemination, particularly when sanitary control measures are insufficient (Britton et al. 1987; Cardoso et al. 2006). Although the dam in the present case had undergone artificial insemination, no epidemiological investigation of the semen, bull, reproductive tract of the dam, or other animals in the herd was performed. Therefore, the route of infection could not be determined, and artificial insemination should be considered only one of several possible sources of exposure. These observations highlight the importance of considering *U. diversum* in the differential diagnosis of bovine abortion regardless of the reproductive management system and reinforce the need for appropriate sanitary monitoring during breeding programs.

Although *U. diversum* infection is more frequently associated with heifers, likely due to reduced prior exposure and limited acquired immunity, abortion occurring in an eight-year-old multiparous cow suggests that additional host and management-related risk factors may contribute to disease expression. The organism is considered part of the bovine genital microbiota and may persist subclinically within the reproductive tract, acting as an opportunistic pathogen when host–microbiota homeostasis is disrupted. Physiological stress, immunomodulation during gestation, or alterations in the uterine microbiota may favor bacterial proliferation and ascending infection, supporting disease development even in previously exposed animals (Andrade et al. 2020).

Osteoarticular involvement has emerged as an uncommon but relevant finding in bovine abortions associated with *Ureaplasma* spp. A case series from the Animal Health Laboratory at the University of Guelph described fetal arthritis in approximately 30% of abortions attributed to this group of organisms, characterized by joint enlargement, cartilage erosion, and synovial alterations (Brooks 2019). Similarly, bilateral lesions affecting the coxofemoral joints represented the only gross abnormality observed in the present fetus, emphasizing the diagnostic value of systematic joint evaluation during fetal necropsy. The identification of subchondral bone necrosis further contributes to the understanding of the disease spectrum, as this lesion has been only sporadically described in association with *U. diversum* (Himsworth et al. 2009; Kerr et al. 2023).

The association between severe multifocal subacute erosive fibrinonecrotizing arthritis with proliferative lymphoplasmacytic synovitis and secondary osteonecrosis, pyogranulomatous pneumonia with bronchus-associated lymphoid tissue (BALT) hyperplasia, and the molecular detection of *U. diversum* in multiple fetal tissues is consistent with an etiological association between the organism and the observed lesions. Pulmonary involvement is considered one of the most consistent pathological findings in fetal infections caused by this microorganism, as previously reported in Brazilian cases (Ribeiro et al. 2025). Although the paraffin-embedded coxofemoral joint sample tested negative by qPCR, this finding should be interpreted with caution because DNA extracted from formalin-fixed, paraffin-embedded (FFPE) tissues is frequently fragmented, which may reduce PCR sensitivity, particularly in samples subjected to prolonged fixation and decalcification. Therefore, a negative molecular result does not necessarily exclude the presence of the organism within the affected joint. Consequently, the lack of direct molecular confirmation within the articular lesion should be considered a limitation of the present study. The concomitant occurrence of pulmonary and articular lesions may suggest hematogenous dissemination within the fetus, although the precise pathogenic mechanisms remain incompletely understood (Santos-Junior et al. 2021).

Molecular confirmation by PCR was essential for establishing the diagnosis, particularly due to the absence of placental tissue for histopathological evaluation. Molecular methods allow accurate differentiation of *U. diversum* from other members of the class Mollicutes and demonstrate higher sensitivity and specificity compared with traditional culture techniques (Santos-Junior et al. 2021). These diagnostic tools are therefore particularly valuable in abortion investigations where sample quality or availability is limited.

The articular lesions observed showed morphological similarities to those reported in humans with mycoplasmal arthritis associated with immunological dysfunction (Franz et al. 1997) and in juvenile calves with mycoplasmal arthritis (Foster 2022). However, these observations are limited to the histomorphological appearance of the lesions and should not be interpreted as evidence of shared pathogenic mechanisms across species or developmental stages. The relatively mild inflammatory infiltrate associated with areas of cartilage necrosis raises the possibility that ischemic injury may have contributed to lesion development, as previously suggested by Himsworth et al. (2009). Nevertheless, the pathogenesis of these lesions remains uncertain, and the contribution of ischemic injury should be regarded as a hypothesis requiring further investigation.

Although placental tissue, which is often critical for confirming the etiology of infectious abortion, was not available for histopathological evaluation, and bacterial isolation was not performed, detailed herd reproductive information, including the occurrence of repeat breeding, infertility, or additional abortions, was also unavailable. Consequently, the epidemiological context of this case could not be comprehensively assessed. Nevertheless, major infectious causes of bovine abortion were investigated and excluded by molecular testing, including BVDV-1, 2 and 3, BoHV-1, BoHV-6, *Leptospira* spp., *B. abortus*, *M. bovis*, and *N. caninum*, all of which yielded negative results. Furthermore, because bacterial culture was not performed, the involvement of other bacterial pathogens associated with fetal arthritis cannot be completely excluded. Therefore, although definitive causality cannot be established based on a single case, the combination of gross findings, histopathological lesions, and the molecular detection of *U. diversum* in multiple fetal tissues provides supportive evidence for an etiological association with the observed lesions.

In conclusion, this case contributes to expanding the currently recognized pathological spectrum of *U. diversum*-associated fetal disease, highlighting the occurrence of severe multifocal subacute erosive fibrinonecrotizing arthritis with proliferative lymphoplasmacytic synovitis and secondary osteonecrosis in conjunction with pulmonary disease. These findings reinforce the importance of systematic articular examination during fetal necropsy and support the inclusion of *U. diversum* in the differential diagnosis of bovine abortion, even in multiparous cows managed under intensive reproductive systems.

## Data Availability

No datasets were generated or analysed during the current study.
